# Combining PSA and PET features to select candidates for salvage lymph node dissection in recurrent prostate cancer

**DOI:** 10.1002/bco2.182

**Published:** 2022-08-04

**Authors:** Carlo A. Bravi, Axel Heidenreich, Nicola Fossati, Giorgio Gandaglia, Nazareno Suardi, Elio Mazzone, Armando Stabile, Vito Cucchiara, Daniar Osmonov, Klaus‐Peter Juenemann, R. Jeffrey Karnes, Alexander Kretschmer, Alexander Buchner, Christian Stief, Andreas Hiester, Peter Albers, Gaëtan Devos, Steven Joniau, Hendrik Van Poppel, Bernhard Grubmüller, Shahrokh Shariat, Derya Tilki, Markus Graefen, Inderbir S. Gill, Alexander Mottrie, Pierre I. Karakiewicz, Francesco Montorsi, Alberto Briganti, David Pfister

**Affiliations:** ^1^ Division of Oncology/Unit of Urology, URI IRCCS Ospedale San Raffaele Milan Italy; ^2^ Department of Urology OLV Ziekenhuis Aalst Aalst Belgium; ^3^ ORSI Academy Ghent Belgium; ^4^ Department of Urology University of Cologne Cologne Germany; ^5^ Department of Urology, Policlinico San Martino Hospital University of Genova Genoa Italy; ^6^ Department of Urology and Pediatric Urology, Campus Kiel University Hospital Schleswig Holstein Kiel Germany; ^7^ Department of Urology Mayo Clinic Rochester Minnesota USA; ^8^ Department of Urology Ludwig‐Maximilians‐University Munich Germany; ^9^ Universitätsklinikum des Saarlandes Homburg Germany; ^10^ Department of Urology, Medical Faculty Heinrich‐Heine‐University Düsseldorf Germany; ^11^ Department of Urology University Hospitals Leuven Leuven Belgium; ^12^ Department of Urology Medical University of Vienna Vienna Austria; ^13^ Institute for Urology and Reproductive Health Sechenov University Moscow Russia; ^14^ Department of Urology University Hospital Hamburg‐Eppendorf Hamburg Germany; ^15^ Martini‐Klinik Prostate Cancer Center University Hospital Hamburg‐Eppendorf Hamburg Germany; ^16^ USC Institute of Urology University of Southern California Los Angeles California USA; ^17^ Cancer Prognostics and Health Outcomes Unit University of Montreal Health Centre Montreal Quebec Canada

**Keywords:** androgen deprivation therapy, metastasis‐directed therapy, neoplasm recurrence, prostate cancer, PSMA PET scan, salvage lymph node dissection

## Abstract

**Objective:**

To evaluate the relationship between pre‐operative PSA value, ^68^Ga‐prostate‐specific‐membrane‐antigen (PSMA) PET performance and oncologic outcomes after salvage lymph node dissection (sLND) for biochemical recurrent prostate cancer (PCa).

**Patients and methods:**

The study included 164 patients diagnosed with ≤2 pelvic lymph‐node recurrence(s) of PCa documented on ^68^Ga‐PSMA PET scan and treated with pelvic ± retroperitoneal sLND at 11 high‐volume centres between 2012 and 2019. Pathologic findings were correlated to PSA values at time of sLND, categorized in early (<0.5 ng/ml), low (0.5–0.99 ng/ml), moderate (1–1.5 ng/ml) and high (>1.5 ng/ml). Clinical recurrence (CR)‐free survival after sLND was calculated using multivariable analyses and plotted over pre‐operative PSA value.

**Results:**

Median [interquartile range (IQR)] PSA at sLND was 1.1 (0.6, 2.0) ng/ml, and 131 (80%) patients had one positive spot at PET scan. All patients received pelvic sLND, whereas 91 (55%) men received also retroperitoneal dissection. Median (IQR) number of node removed was 15 (6, 28). The rate of positive pathology increased as a function of pre‐operative PSA value, with highest rates for patients with pre‐operative PSA > 1.5 ng/ml (pelvic‐only sLNDs: 84%; pelvic + retroperitoneal sLNDs: 90%). After sLND, PSA ≤ 0.3 ng/ml was detected in 67 (41%) men. On multivariable analyses, pre‐operative PSA was associated with PSA response (*p* < 0.0001). There were 51 CRs after sLND. After adjusting for confounders, we found a significant, non‐linear relationship between PSA level at sLND and the 12‐month CR‐free survival (*p* < 0.0001), with the highest probability of freedom from CR for patients who received sLND at PSA level ≥1 ng/ml.

**Conclusions:**

In case of PET‐detected nodal recurrences amenable to sLND, salvage surgery was associated with the highest short‐term oncologic outcomes when performed in men with PSA ≥ 1 ng/ml. Awaiting confirmatory data from prospective trials, these findings may help physicians to optimize the timing for ^68^Ga‐PSMA PET in biochemical recurrent PCa.

## 1 INTRODUCTION

Patients with prostate cancer treated with radical prostatectomy have a risk of biochemical relapse that ranges between 27% and 53%.^1^, ^2^ In this regard, modern imaging techniques are getting more important for further treatment decision making for men who experience a PSA rise after primary treatment. At present time, the most frequently performed imaging is ^68^Ga‐prostate‐specific‐membrane‐antigen (PSMA) PET as it ensures demonstrably superior diagnostic accuracy as compared with the formally used ^11^C‐choline tracer.^3^, ^4^ This is mainly sustained by histology‐correlated analyses where ^68^Ga‐PSMA PET showed higher accuracy than ^11^C‐choline for the detection of recurrent disease^4^, ^5^, ^6^, ^7^ and thus should be considered the first choice.^8^

In men with recurrent prostate cancer after primary treatment, current guidelines recommend the performance of ^68^Ga‐PSMA PET at PSA values >0.2 ng/ml if a change in treatment can be expected. However, positive nuclide uptake of ^68^Ga‐PSMA can be expected in approximately 50% of patients at these low PSA values,^3^, ^9^, ^10^ and as such, it is often difficult to distinguish candidates for metastasis‐directed therapies (MDTs) from patients with either non‐metastatic disease or from those who might need systemic treatment.^11^, ^12^, ^13^

Salvage lymph node dissection (sLND) is among possible MDT options for men with node‐only recurrence(s) after radical prostatectomy. Despite good short‐term biochemical control rates,^14^ long‐term oncologic outcomes are somewhat disappointing as a single treatment option,^15^ underlining the importance of adequate patient selection in order to maximize the potential benefit from sLND.^16^, ^17^ Whereas several prognostic tools have been proposed to identify the optimal candidates to this surgical procedure,^16^ sLND is frequently performed as an individual treatment approach on the basis of what is shown on re‐staging imaging. Thus, it is paramount to optimize the timing for ^68^Ga‐PSMA PET in case of PSA rise after primary treatment, a delicate balance between higher detection rate and risk of missing the therapeutic window. For this reason, we assessed the relationship between PSA level at sLND, ^68^Ga‐PSMA PET scan feature and oncologic outcomes with the aim of identifying the patients who have benefited the most from sLND and thus optimize the timing for ^68^Ga‐PSMA PET in biochemical recurrent prostate cancer.

## 2 METHODS

We analysed data of 283 patients with up to two pelvic‐only nodal recurrence(s) after radical prostatectomy detected by ^68^Ga‐PSMA PET who underwent sLND at 11 tertiary referral centres between 2012 and 2019. For all patients, the interval between ^68^Ga‐PSMA PET and sLND was no longer than 3 months. To exclude other sites of recurrence, pre‐operative imaging included abdominal CT scan, whole‐body MRI or bone scan using technetium Tc^99^m methylene diphosphonate according to the standard of care at each treating institution. We excluded patients with PSA level at sLND higher than 4 ng/ml (*n* = 55) and those with missing covariates [androgen deprivation therapy (ADT) administration at PET scan, *n* = 39; first PSA after sLND, *n* = 25], resulting in 164 patients eligible for the analyses.

### 2.1 Surgical technique and follow‐up

All patients underwent a template lymph node dissection with a minimum of resection of lymph nodes surrounding the external, internal and obturator artery, as well as the common iliac artery as described earlier.^16^ If clinically suspicious or in case of positive frozen sections, the resection field was extended to the pararectal, presacral and retroperitoneal region. Follow‐up consisted of PSA testing 1 month after surgery, and, subsequently, further measurements were performed at time intervals of 3–6 months. PSA responders were defined by PSA decrease after sLND ≤0.3 ng/ml.^18^ The administration of additional treatments after sLND was left at the discretion of each surgeon. Clinical recurrence (CR) after sLND was defined as positive conventional imaging in presence of a rising PSA, with patients censored on the date of last evidence of freedom from CR.

### 2.2 Statistical analyses

Our statistical analyses included several steps. First, we assessed descriptive characteristics of our cohort. Second, we correlated pathologic findings in different resected fields during sLND with pre‐operative PSA values. Third, the probability of freedom from CR after sLND [and corresponding 95% confidence interval (CI)] were calculated using Kaplan–Meier analyses. Fourth, multivariable regression models were utilized to assess the association between PSA at sLND and oncologic outcomes after sLND, namely, PSA response and CR‐free survival after sLND. The adjustment for case mix included the following variables that were selected a priori: When PSA response was the outcome of interest, the model included ISUP group at RP (≥4 vs. ≤5), ADT administration at PET scan (yes vs. no) and number of positive spots at PET scan (1 vs. 2), whereas the analyses for CR‐free survival also included variables known after surgery such as PSA response after sLND (yes vs. no), administration of ADT within 6 months from sLND (yes vs. no), administration of RT within 6 months from sLND (yes vs. no). In this latter model, we utilized landmark analyses with follow‐up starting 6 months after sLND. PSA at sLND was included as a non‐linear term using restricted cubic splines with knots at quartiles. Because data from different institutions are correlated, we incorporated institution clustering in our analysis using the *cluster* option in Stata statistical software. Compared with the main cohort that included 164 patients, seven men were not included in the survival analyses due to missing data. Finally, to visualize our findings, the probability of freedom from CR 1 year after sLND was calculated for an average patient, that is, by setting variables at the mean.

## 3 RESULTS

Patient characteristics are described in Table 1. At RP, 24 (15%) patients had ISUP Group 5 tumour, whereas 22 (13%) had nodal involvement. A total of 151 (92%) patients were free of androgen deprivation therapy at time of ^68^Ga‐PSMA PET, and approximately four in five patients had one positive spot on imaging. Median [interquartile range (IQR)] PSA and age at sLND were 1.1 (0.6, 2.0) ng/ml and 67 (62, 71) years, respectively. Median (IQR) number of resected nodes at sLND was 15 (6, 28). In the whole study population, final pathology was positive for 131 (80%) patients.

**TABLE 1:** Descriptive characteristics of 164 patients with nodal recurrence of PCa after radical prostatectomy detected by ^68^Ga‐PSMA PET scan who underwent sLND

	Overall population (*n* = 164; 100%)
Pathologic ISUP group	
≤4	140 (85%)
5	24 (15%)
pT stage	
pT2	52 (32%)
pT3a	60 (37%)
pT3b–pT4	44 (27%)
Unknown	8 (4%)
pN stage	
pN0	110 (67%)
pN1	22 (13%)
pNx	26 (16%)
Unknown	6 (4%)
Positive surgical margins	
No	104 (63%)
Yes	41 (25%)
Unknown	19 (12%)
RT administration after RP	88 (54%)
Years between RP and sLND	4 (2, 6)
Age at sLND, years	67 (62, 71)
PSA level at sLND, ng/ml	1.1 (0.6, 2.0)
ADT administration at PET scan	13 (8%)
Number of positive spots at PET scan	
1	131 (80%)
2	33 (20%)

Abbreviations: ADT, androgen deprivation therapy; ISUP, International Society of Urologic Pathology; PCa, prostate cancer; PSMA, prostate‐specific membrane antigen; RP, radical prostatectomy; RT, radiation therapy; sLND, salvage lymph node dissection.

Table 2a,b correlates pathologic findings in different resected fields with pre‐operative PSA values. Among patients who receive sLND only in the pelvis, final pathology was positive for prostate cancer in 53/73 (73%) men. By contrast, 78/91 (86%) patients receiving pelvic and retroperitoneal dissection had positive pathology. We found that findings on final pathology were influenced by PSA level at sLND, with increasing rate of positive pathology for higher PSA level at sLND. For instance, among patients receiving pelvic‐only sLND, the rate of positive pathology increased from 55% at early (<0.5 ng/ml) PSA values to more than 80% in case of PSA value >1.5 ng/ml (Table 2a). Similarly, the rate of positive pathology for men receiving both pelvic and retroperitoneal dissection increased as a function of pre‐operative PSA level, with highest percentages for men who received surgery at PSA values equal or greater than 1 ng/ml (Table 2b).

**TABLE 2:** Pathologic findings and biochemical response rates of patients who underwent (a) pelvic‐only and (b) pelvic + retroperitoneal sLND for ^68^Ga‐PSMA PET‐detected nodal recurrence(s) of PCa after radical prostatectomy, stratified by pre‐operative PSA level

(a) Pelvic‐only sLND (*n* = 73)	
Final pathology positive for PCa	
No	20 (27%)
Yes	53 (73%)
Final pathology positive for PCa	
PSA < 0.5 ng/ml	6/11 (55%)
PSA 0.5–0.99 ng/ml	9/14 (64%)
PSA 1–1.5 ng/ml	11/16 (69%)
PSA > 1.5 ng/ml	27/32 (84%)
PSA response after sLND (≤0.3 ng/ml)	
PSA < 0.5 ng/ml	9/11 (82%)
PSA 0.5–0.99 ng/ml	4/14 (29%)
PSA 1–1.5 ng/ml	5/16 (31%)
PSA > 1.5 ng/ml	8/32 (25%)

(b) Pelvic + retroperitoneal sLND (*n* = 91)	
Final pathology positive for PCa	
No	13 (14%)
Yes	78 (86%)
Final pathology results among pelvic + retroperitoneal sLND	
Negative pathology in both pelvis and retroperitoneum	13 (14%)
Pelvis −, retroperitoneum +	12 (13%)
Pelvis +, retroperitoneum −	36 (40%)
Pelvis +, retroperitoneum +	10 (11%)
Positive pathology, but unknown location	20 (22%)
Final pathology positive for PCa	
PSA < 0.5 ng/ml	11/17 (65%)
PSA 0.5–0.99 ng/ml	25/28 (89%)
PSA 1–1.5 ng/ml	15/16 (94%)
PSA > 1.5 ng/ml	27/30 (90%)
PSA response after sLND (≤0.3 ng/ml)	
PSA < 0.5 ng/ml	11/17 (65%)
PSA 0.5–0.99 ng/ml	18/28 (64%)
PSA 1–1.5 ng/ml	7/16 (44%)
PSA > 1.5 ng/ml	5/30 (17%)

Abbreviations: PCa, prostate cancer; sLND, salvage lymph node dissection.

**FIGURE 1:**
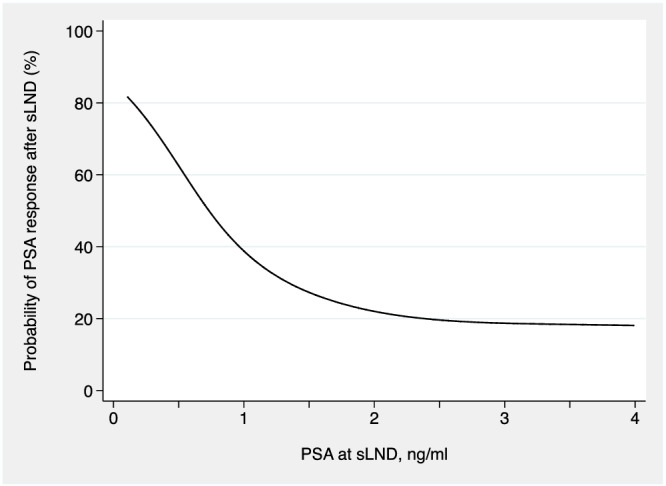
Relationship between PSA at salvage lymph node dissection and probability of PSA response (≤0.3 ng/ml) after surgery

A total of 67 (41%) patients had PSA response after surgery. As shown in Table 2, despite higher rates of positive pathology in patients with higher pre‐operative PSA, the percentage of PSA response after surgery was lowest for patients with pre‐operative PSA > 1.5 ng/ml. After adjusting for confounders, we found a significant, non‐linear relationship between PSA at sLND and the probability of PSA response after surgery (*p* < 0.0001). As shown in Figure 1, the probability of having a PSA ≤ 0.3 ng/ml after sLND decreased as a function of pre‐operative PSA and plateaued after 1 ng/ml.

**FIGURE 2:**
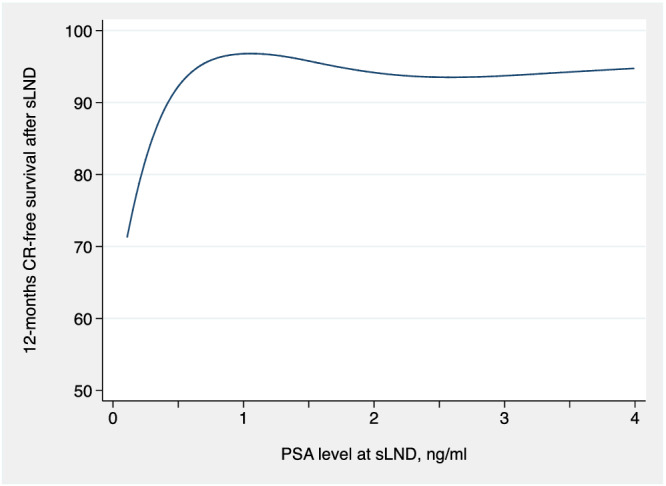
Probability of freedom from clinical recurrence 12 months after sLND over PSA level at sLND

A total of 51 patients experienced CR after sLND. Median (IQR) follow‐up for survivors was 12 (5, 25) months. The 12‐month CR‐free survival was 84% (95% CI: 76%, 89%). On multivariable analyses, we found a significant, non‐linear association between PSA value at sLND and the risk of CR after surgery (*p* < 0.0001). Figure 2 describes the 12‐month CR‐free survival over pre‐operative PSA value. As can be seen, the probability of being free from CR 12 months from surgery was highest for patients who received sLND at PSA ≥ 1 ng/ml, whereas sLND performed at lower PSA values did not translate in better CR‐free survival.

## 4 DISCUSSION

In this study, we assessed the relationship between PSA level at sLND, ^68^Ga‐PSMA PET scan performance and oncologic outcomes after sLND. Although patients receiving sLND at low PSA level had higher rate of PSA response after surgery, this did not automatically translate into better oncologic outcomes. On the contrary, patients who were treated at PSA ≥ 1 ng/ml had the highest probability of freedom from CR 1 year after sLND.

In men with biochemical recurrence after radical prostatectomy, ^68^Ga‐PSMA PET scan allows for the detection of possible metastases at low PSA values. This goes with the common assumption that the sooner metastases are detected and salvage treatment is performed, the greater are the chances of oncologic benefit for the patients. However, at low PSA levels, imaging might be negative in up to half of the patients,^19^, ^20^ and small metastases are often visualized in atypical localizations that are hard to find intra‐operatively. Our data showed that, although the rate of pathologically proven metastases increased with pre‐operative PSA level, this did not always translate in higher rates of PSA response after surgery. Moreover, men receiving sLND at PSA level below 1 ng/ml had higher rates of PSA response after surgery, but still, this did not ensure the best oncologic outcomes for this subgroup of patients. As such, our results suggest that there are number of factors involved in the delicate balance between imaging performance, surgical resection and oncologic results after sLND.

A promising implementation that has the potential to fill this gap is radio‐guided surgery (RGS). Maurer et al. first described the technique in 2015^21^ and, more recently, published their experience in a series of 31 men.^22^ All lesions described on ^68^Ga‐PSMA PET were removed, and metastases were found in 58/132 resected specimens. No specimen was false positive, yielding a positive predictive value of 100%, but 12 specimens were false negative for metastases as small as 2 mm. The same investigators also correlated intra‐operative performance with biochemical response after surgery in a larger cohort of men treated with RGS for node‐only recurrent prostate cancer.^23^ Among 121 men receiving surgery, final pathology was positive in 99% of patients, with a 66% rate of complete biochemical response (PSA < 0.2 ng/ml). Similar results were described in a retrospective comparison of template resection only versus RGS: Although RGS had a 100% detection rate, final pathology was negative in approximately one in three patients who received a template resection only.^24^ This allowed for an almost doubled rate of PSA decline >50% in the RGS group (92% vs. 29%). By contrast, other investigators described worse results after RGS, with a true positive and false negative rate of 61% and 37%, respectively.^25^ However, given the quite high mean PSA level at sLND in this cohort (7.9 ng/ml), it is possible that the worse performance of RGS might be a consequence of patient selection rather than poor results of the gamma probe itself. Taken together, RGS is a promising technique that has the potential to reduce imaging underestimation and optimize surgical resection, but, unfortunately, it is rather new and not frequently available yet. Among other implementations that might be valuable instruments for surgical guidance, near‐infrared fluorescence with indocyanine green has been investigated for robotic urologic surgery, with promising results.^26^, ^27^

Our data entails a second relevant point for clinical practice. Unlike patients receiving salvage radiotherapy after radical prostatectomy, who have demonstrably more favourable outcomes when treated at very early PSA levels,^28^ this seems not to be the case for candidates to sLND. In fact, we found that patients receiving sLND at low PSA level had worse oncologic outcomes than men treated at PSA ≥ 1 ng/ml. Because the success of MDT is highly influenced by imaging performance, it is plausible that suboptimal outcomes for patients receiving sLND at PSA < 1 ng/ml might be a result of disease underestimation, a well‐known limitation of re‐staging imaging.^3^ In this regard, several prior studies correlating PET scan findings with final pathology results showed that imaging performance for lymph node metastases <3 mm is suboptimal.^6^, ^29^, ^30^ Thus, a ^68^Ga‐PSMA PET performed at very early PSA values may have higher chances of false negative findings, and thus, it may miss additional nodal metastases, and/or it may diagnose oligometastatic prostate cancer although the disease is already systemic. In both these scenarios, the potential benefit associated with MDT is inevitably limited. This is compatible with our findings as they suggest that, in candidates to sLND, there might be a certain early temporal window in which surgery performed at low PSA levels might translate in higher rate of PSA response after surgery, whereas the oncologic benefit of sLND might not be maximized. If replicated, our results might have relevant implications for clinical practice as they suggest that, in men with biochemical recurrence after radical prostatectomy, re‐staging PET scan might be performed at PSA of approximately 1 ng/ml.

Our study has several limitations. First, no control group including men managed with either observation or systemic treatment was available. Moreover, the use of additional treatments after sLND was left at the discretion of physicians. As such, it is hard to extrapolate the relative contribution of sLND and subsequent treatments to oncologic outcomes given possible patient selection biases. However, the role of a multi‐modal approach was confirmed at multivariable analyses accounting for all possible significant confounders. Moreover, we cannot exclude residual confounding from known and unknown variables. For instance, the multi‐institutional data collection did not account for central specimen review or for surgical experience of each treating surgeon. Because there is evidence that these factors might influence results and outcomes after surgery,^31^, ^32^ it is plausible that the inclusion of such features might affect our results. Also, we have to acknowledge that our finding of higher rate of positive pathology after sLND with higher PSA values might be a consequence of higher tumour volume (and larger lymph node metastases). Despite these limitations, our study represents the first study assessing the relationship between PSA level at sLND, ^68^Ga‐PSMA PET scan performance and oncologic outcomes after sLND.

## 5 CONCLUSIONS

In men who received sLND for node‐only recurrence after radical prostatectomy, a delicate balance exists between pre‐operative PSA level, imaging performance and oncologic outcomes after surgery. Despite higher rates of PSA response after sLND, patients receiving sLND at lower PSA levels had worse CR‐free survival than those treated at PSA level ≥1 ng/ml. Awaiting results from prospective studies, these findings might help physicians to optimize the timing for re‐staging imaging in biochemical recurrent prostate cancer.

## AUTHOR CONTRIBUTIONS

Study concept and design: Pfister, Bravi, Heidenreich. Acquisition of data: Mazzone, Stabile, Cucchiara, Devos, Gandaglia, Fossati. Drafting of the manuscript: Bravi, Pfister. Critical revision of the manuscript for important intellectual content: Bravi, Briganti, Montorsi, Karakiewicz, Mottrie, Karnes, Kretschmer, Albers, Joniau, Van Poppel, Shariat, Tilki, Graefen, Gill, Suardi, Osmonov, Juenemann, Buchner, Stief, Hiester, Grubmüller.

## Supporting information


**Data S1.** Supporting Information
